# A Joint Fault Diagnosis Scheme Based on Tensor Nuclear Norm Canonical Polyadic Decomposition and Multi-Scale Permutation Entropy for Gears

**DOI:** 10.3390/e20030161

**Published:** 2018-03-03

**Authors:** Mao Ge, Yong Lv, Cancan Yi, Yi Zhang, Xiangjun Chen

**Affiliations:** 1 Key Laboratory of Metallurgical Equipment and Control Technology, Wuhan University of Science and Technology, Wuhan 430081, China; 2 Hubei Key Laboratory of Mechanical Transmission and Manufacturing Engineering, Wuhan University of Science and Technology, Wuhan 430081, China; 3 Zhejiang Provincial Special Equipment Inspection Research Institute, Hangzhou 314415, China

**Keywords:** tensor decomposition, nuclear norm, convex optimization, multi-scale permutation entropy, gear fault classification

## Abstract

Gears are key components in rotation machinery and its fault vibration signals usually show strong nonlinear and non-stationary characteristics. It is not easy for classical time–frequency domain analysis methods to recognize different gear working conditions. Therefore, this paper presents a joint fault diagnosis scheme for gear fault classification via tensor nuclear norm canonical polyadic decomposition (TNNCPD) and multi-scale permutation entropy (MSPE). Firstly, the one-dimensional vibration data of different gear fault conditions is converted into a three-dimensional tensor data, and a new tensor canonical polyadic decomposition method based on nuclear norm and convex optimization called TNNCPD is proposed to extract the low rank component of the data, which represents the feature information of the measured signal. Then, the MSPE of the extracted feature information about different gear faults can be calculated as the feature vector in order to recognize fault conditions. Finally, this researched scheme is validated by practical gear vibration data of different fault conditions. The result demonstrates that the proposed scheme can effectively recognize different gear fault conditions.

## 1. Introduction

Gears are key components in rotation machinery [1], whose operating performance can directly affect the health status of the entire system. When the system stops work due to gear faults, such as circular pitch faults, abrasion faults, and broken teeth faults, heavy productivity and economic losses will occur. Consequently, it is of great practical significance to achieve fault diagnosis of gear faults. So far, many fault diagnosis technologies for gear fault diagnosis has been proposed, such as monitoring techniques based on vibration and noise [2], oil analysis methods [3], and non-destructive testing techniques [4]. Vibration signal analysis may be the most useful technology in light of its huge technological advantages. In general, the gear vibration signal is composed of multi-components, where the meshing frequencies of each pair of gears are the dominant characteristic [5]. When fault occurs, the meshing frequencies and their multiple frequencies are modulated by the rotational frequency of the fault gears, thereby generating amplitude-modulated and frequency-modulated (AM-FM) signals [6]. Moreover, the working environment of rotation machinery is relatively poor, the collected signals usually contain massive noise, and the fault component may be masked by the strong noise. Commonly, the gear fault diagnosis of different working conditions includes a fault feature extraction process and a fault recognition process [7], where feature extraction is the essential step [8].

Many scholars have proposed different fault diagnosis methods for extracting fault information from signals in the time domain or the frequency domain. The generalized demodulation time–frequency analysis method [9] is suitable for dealing with multi-component and AM-FM signals, which can decompose the signal into a set of single-component signals, whose instantaneous frequencies have physical meaning. However, the choice of the phase function is a difficult problem. Analogously, the local mean decomposition (LMD) method [10] can decompose any complicated multi-component signal into a series of the product of an amplitude envelope signal and a purely frequency-modulated signal, but it still possesses some problems such as modal aliasing. Wavelet transform (WT) [11], which can decompose the signal into low-frequency components and high-frequency components sub-band, has been widely used in mechanical equipment fault diagnosis. However, the choice available of decomposition level and wavelet basis function has a great effect on the analysis results. Transforming the one-dimensional signal to a high-dimensional phase space, the attractor can view the dynamic behavior of the original signal, which facilitates the separation of useful multi-component features and noise components [12]. As a powerful method for analyzing a real-valued time series in matrix space, singular spectrum analysis (SSA) [13] can decompose a signal into interpretable components, such as useful signal components and noise. However, SSA may not work well for a non-stationary signal, and selecting a desired subgroup of eigenvalues should receive considerable attention [14].

For the drawbacks of standard flat matrix models in dealing with non-linear and non-stationary signals, high-dimensional space contains more abundant information than matrix space [15]. Therefore, converting the signal into high-dimensional space may explicitly show the properties of the signal [14]. As the natural way of representing high-dimensional data, tensors (multi-dimensional arrays) can efficiently maintain the intrinsic structural characteristics of data [15]. In fact, tensors can be seen as the generalization of matrices (two-dimensional arrays), and signal processing techniques using tensor decompositions can extract more latent features of data than matrix-based methods [16]. Tensor decomposition models can be theoretically divided into two categories: canonical polyadic decomposition (CPD) [17] and tucker decomposition [18]. CPD is employed in this paper, as it can decompose any complex multi-component signal data into a sum of rank-one sub-tensors composed of factor matrices [19], which may represent characteristic information. Due to the deficiency of traditional iterative least squares algorithm for CPD (CPD-ALS) in handling non-linear, non-stationary, and strong noise background signals, a new tensor canonical polyadic decomposition method based on nuclear norm [20] and convex optimization [21,22], named TNNCPD, is proposed in this paper. The matrix nuclear norm, which is defined as the sum of all singular values, can solve the non-convex problem of the rank constraint [23]. Nuclear norm minimization method can obtain an accurately approximate low-rank matrix of the original matrix signal data, which represent characteristic information, and has a positive effect on the image signal processing [24]. Therefore, we extend the matrix nuclear norm to the three-dimensional tensor. Firstly, the one-dimensional gear vibration signal data is converted into three-dimensional tensor data via segmentation [14,15] and the Löwner matrix [25]. Then, a tensor nuclear norm definition is given by applying a nuclear norm minimum constraint on the approximate tensor in each iteration, and the minimum problem can be transformed into a convex optimization problem via imposing convex relaxation constraint on the factor matrices. Moreover, the alternating iterative decline and singular value threshold [26] are employed to obtain globally optimal low-rank factor matrices, which contain the characteristic components of the original signal. Lastly, the proposed method is utilized to extract the fault feature of different gear working condition signals. The calculated results demonstrate that the TNNCPD method is effective.

Followed by the fault recognition process, the feature information of original signals can be obtained through the TNNCPD model. Since the measured signal is composed of multi-components due to the interaction between machine components, characterizing the characteristic distribution of gear fault signal on a single scale may be insufficient [27]. As an effective approach to evaluate the complexity of a time series in different scales, multi-scale permutation entropy (MSPE) [28] has become an indicator for the dynamic behavior of different signal fault patterns [29]. Therefore, MPSE is used as the gear fault pattern recognizer by solving the multi-scale permutation entropy vector of the extracted feature component and setting it as the input vector of the back propagation (BP) neural network [30] to perform fault classification. In summary, a joint fault diagnosis scheme via TNNCPD and MSPE is presented and has been applied to gear fault classification. The results illustrate that the proposed scheme can accurately recognize different gear working conditions. Thus, the proposed scheme can be used for gear fault diagnosis.

### 1.1. Notation

We use lowercase letters that represent a scalar, such as $x_{1}$, bold and lowercase letters for a vector, such as $x\in {\mathbb{R}}^{n}$, and the upper case and bold letters for a matrix, such as $X\in {\mathbb{R}}^{m\times n}$. $\sigma_{i}(X)$ represents the *i*-th largest singular value of X, and the matrix nuclear norm is defined as ${\left\|X\right\|}_{*}:=\sum \sigma_{i}(X)$. A three-dimensional tensor is represented as $\chi \in {\mathbb{R}}^{I_{1}\times I_{2}\times I_{3}}$, while the *k*-mode unfold matrix of χ can be denoted as $X_{\left(k\right)},k=1,2,3$. The Frobenius norm of matrix and the tensor is defined as ${\left\|X\right\|}_{F}:={\left(\sum {\left|x_{ij}\right|}^{2}\right)}^{0.5}$ and ${\left\|\chi \right\|}_{F}={\left(\sum {\left|x_{i_{1}i_{2}i_{3}}\right|}^{2}\right)}^{0.5}$, respectively.

### 1.2. Organization

This paper is organization as follows. The theory of TNNCPD is put forward in Section 2; Section 3 illustrates a joint fault diagnosis scheme based on TNNCPD and MSPE in detail; the measured gear fault data is analyzed in Section 4, and conclusions are drawn in Section 5.

## 2. Tensor Nuclear Norm Canonical Polyadic Decomposition (TNNCPD)

### 2.1. Tensor Canonical Polyadic Decomposition

#### 2.1.1. The Tensorization of One-Dimensional Vibration Signal Data

**Figure 1:**
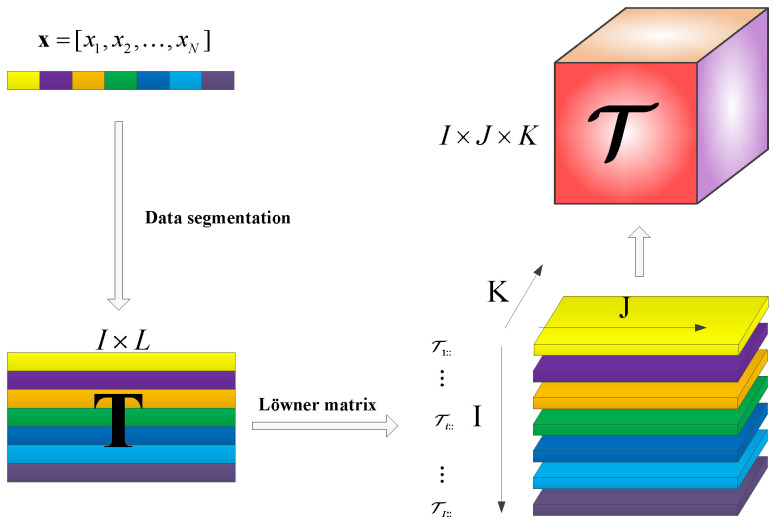
Löwner matrix-based tensor construction process.

The segmentation [14,15] and Löwner matrix [25] is employed to convert the measured one-dimensional gear vibration signal data $x\in {\mathbb{R}}^{N}$ into a three-dimensional tensor data $T\in {\mathbb{R}}^{I\times J\times K}$. The process is called tensorization and the tensor construction process is demonstrated in Figure 1.

The tensor construction process contains two steps. Firstly, x is cut into a matrix $T\in {\mathbb{R}}^{I\times L}$ with a non-overlapping window length L. To improve the computing speed, we select the window length as $L=[\sqrt{N}]$.

(1) $$T=\left(\begin{array}{cccc} x_{1} & x_{2} & \ldots & x_{L}\\ x_{\left(L+1\right)} & x_{\left(L+2\right)} & \cdots & x_{2L}\\ \vdots & \vdots & \ddots & \vdots \\ x_{(I-1)\times L+1} & x_{(I-1)\times L+2} & \cdots & x_{N} \end{array}\right).$$

Then, each row of T is mapped into a Löwner matrix form with the corresponding horizontal slice $T_{i::}\in {\mathbb{R}}^{J\times K}$.

(2) $$T_{i::}=\left[\begin{array}{cccc} \frac{t_{i,1}-t_{i,2}}{1-2} & \frac{t_{i,1}-t_{i,4}}{1-3} & \cdots & \frac{t_{i,1}-t_{i,L}}{1-L}\\ \frac{t_{i,3}-t_{i,2}}{3-2} & \frac{t_{i,3}-t_{i,4}}{3-4} & \cdots & \frac{t_{i,3}-t_{i,L}}{3-L}\\ \vdots & \vdots & \ddots & \vdots \\ \frac{t_{i,L-1}-t_{i,2}}{(L-1)-2} & \frac{t_{i,L-1}-t_{i,4}}{(L-1)-4} & \cdots & \frac{t_{i,L-1}-t_{i,L}}{(L-1)-L} \end{array}\right],\;1\leq i\leq I.$$

#### 2.1.2. Tensor Canonical Polyadic Decomposition Based on the Least-Squares Algorithm (CPD-ALS)

**Figure 2:**
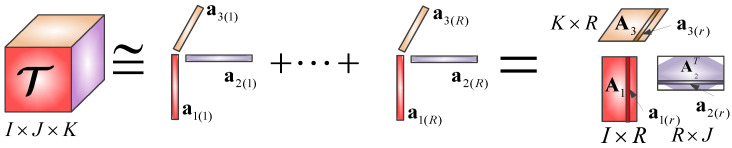
The canonical polyadic decomposition (CPD) of a three-dimensional Rank-*R* tensor.

CPD can decompose a tensor T as a sum of *R* rank-1 sub-tensors, which is defined as an outer product of three vectors $\chi_{r}=a_{1(r)}⚬a_{2(r)}⚬a_{3(r)},\;1\leq r\leq R$. The rank of T is defined as the smallest value *R* [16].

(3) $$T=\tilde{T}+\varepsilon =\sum_{r=1}^{R}a_{1(r)}⚬a_{2(r)}⚬a_{3(r)}+\varepsilon =〚A_{1},A_{2},A_{3}〛+\varepsilon$$

where $A_{1}\in {\mathbb{R}}^{I\times R},\;A_{2}\in {\mathbb{R}}^{J\times R},\;A_{3}\in {\mathbb{R}}^{K\times R}$ are factor matrices, which represent the principal components in each direction, $\tilde{T}\in R^{I\times J\times K}$ is the approximate tensor with a size identical to T, and $\varepsilon \in R^{I\times J\times K}$ is the residual tensor. Figure 2 depicts the CPD of a three-dimensional rank-*R* tensor.

The main goal of CPD is how to effectively solve factor matrices. The traditional method concentrated on minimizing the problem of Equation (4) [18].

(4) $$\min_{A,B,C}\frac{1}{2}\|T-〚A_{1},A_{2},A_{3}〛{\|}_{F}^{2}.$$

The CPD-ALS algorithm [18] is a well-known solution for Equation (4). Firstly, the factor matrices are initialized using the random matrix method, then an iterative process as Equation (5) is applied until Equation (4) converges or reaches the presupposed iteration number; thus, the *R* rank-1 sub-tensors as well as the factor matrices can be obtained.

(5) $$A_{1}^{n}=T_{\left(1\right)}{\left[{\left(A_{2}^{n-1}⊙A_{3}^{n-1}\right)}^{T}\right]}^{†},\;A_{2}^{n}=T_{\left(2\right)}{\left[{\left(A_{3}^{n-1}⊙A_{1}^{n}\right)}^{T}\right]}^{†},\;A_{3}^{n}=T_{\left(3\right)}{\left[{\left(A_{2}^{n}⊙A_{1}^{n}\right)}^{T}\right]}^{†}.$$

CPD-ALS is suitable for linear or high SNR signal processing. However, it is incapable of handling the non-linear and non-stationary signals, such as fault signals with strong background noise. In the following section, we propose the new tensor CPD method based on nuclear norm and convex optimization.

### 2.2. Tensor Nuclear Norm Canonical Polyadic Decomposition (TNNCPD)

#### 2.2.1. Matrix Nuclear Norm

The classical theory of the matrix low rank approximation supports that a matrix data $A\in {\mathbb{R}}^{m\times n}$ can be decomposed into a low-rank component $L\in {\mathbb{R}}^{m\times n},\;r(L)<r(A)$ and a sparse component $S\in {\mathbb{R}}^{m\;\times \;n},\;r(S)\ll r(A)$ [31]. In signal processing, the low-rank component represents the inherent characteristics of signal and the sparse component captures the perturbations or additional noise [31].

(6) A=L+S.

The main difficulty is effectively obtaining the low rank component, L, which can be expressed as a rank constraint problem [23]:

(7) $$\begin{array}{l} \underset{L}{\min}\frac{1}{2}{\left\|L-A\right\|}_{F}^{2}\\ s.t.\;\mathrm{rank}(L)\leq r \end{array}.$$

Solving Equation (7) is difficult because the rank constraint is not convex; a good way of solving this problem is exerting a nuclear norm constraint ${\left\|\cdot \right\|}_{*}$ to the low-rank component, L. Thus, Equation (7) can be turned into a convex optimization problem [32]:

(8) $$\begin{array}{c} \underset{L}{\min}\frac{1}{2}{\left\|L-A\right\|}_{F}^{2}\\ s.t.\;{\left\|L\right\|}_{*}\leq c \end{array}.$$

An accurately approximate low-rank matrix L of original matrix A can be obtained via solving Equation (8), which may represent the characteristic components of the signal.

#### 2.2.2. Tensor Canonical Polyadic Decomposition Method Based on Nuclear Norm and Convex Optimization

Duo to the great effect of matrix nuclear norm in signal feature extraction, we extend the matrix nuclear norm to the three-dimensional tensor. The tensor can be seen as the generalization of the matrix. Similar to the matrix condition, we assume that a three-dimensional tensor data, T, also consists of a low-rank sub-tensor $T=〚A_{1},A_{2},A_{3}〛$ and the sparse sub-tensor ε. Therefore, the approximate tensor $\tilde{T}$, which is composed of three low-rank factor matrices, can represent the characteristic components of the data. The focus of the research is how to accurately extract the low-rank factor matrices. We introduce the nuclear norm to the tensor CPD model by applying the nuclear norm constraint on the approximate tensor:

(9) $$\begin{array}{c} \underset{\tilde{T}}{\min}\frac{1}{2}{\left\|\tilde{T}-T\right\|}_{F}^{2}\\ s.t.\;{\left\|\tilde{T}\right\|}_{*}\leq c \end{array}.$$

We propose a definition of the tensor nuclear norm as follows:

(10) $${\left\|\tilde{T}\right\|}_{*}=\frac{1}{3}\sum_{i=1}^{3}{\left\|{\tilde{T}}_{\left(i\right)}\right\|}_{*}.$$

Equation (10) is the average of the nuclear norm of the three unfolded matrices. As the non-independent relationship among unfolded matrices, solving Equation (10) is a challenge. For this problem, we use the factor matrices to replace $\tilde{T}$; thus, a nuclear norm constraint around the factor matrices can be obtained:

(11) $$\begin{array}{l} \underset{\tilde{T}}{\min}\frac{1}{2}{\left\|\tilde{T}-T\right\|}_{F}^{2}=\frac{1}{6}\underset{\tilde{T}}{\min}\sum_{i=1}^{3}{\left\|{\tilde{T}}_{\left(i\right)}-T_{\left(i\right)}\right\|}_{F}^{2}\\ s.t.\;\frac{1}{3}\sum_{i=1}^{3}{\left\|A_{i}\right\|}_{*}\leq c \end{array}$$

where the relationship between unfolded matrices and factor matrices in Equation (11) are shown as

(12) $${\tilde{T}}_{\left(1\right)}=A_{1}{\left(A_{2}⊙\;A_{3}\right)}^{T},\;{\tilde{T}}_{\left(2\right)}=A_{2}{\left(A_{3}⊙\;A_{1}\right)}^{T},\;{\tilde{T}}_{\left(3\right)}=A_{3}{\left(A_{2}⊙\;A_{1}\right)}^{T}.$$

Equation (11) is still difficult to solve because of the constraints among factor matrices. Therefore, we introduce three independent variable matrices $Y_{1},Y_{2},Y_{3}$ as follows:

(13) $$\begin{array}{l} \frac{1}{6}\underset{Y_{1},A_{i}}{\min}\sum_{i=1}^{3}{\left\|Y_{i}-T_{\left(i\right)}\right\|}_{F}^{2}\\ s.t.\;\frac{1}{3}\sum_{i=1}^{3}{\left\|Y_{i}\right\|}_{*}\leq d\\ Y_{1}={\tilde{T}}_{\left(1\right)},\;Y_{2}={\tilde{T}}_{\left(2\right)},\;Y_{3}={\tilde{T}}_{\left(3\right)} \end{array}.$$

The last line of Equation (13) is a tight constraint. We apply a convex relaxation constraint to it:

(14) $$\underset{Y_{i},A_{i}}{\min}\frac{1}{6}\sum_{i=1}^{3}\alpha_{i}{\left\|Y_{i}-T_{\left(i\right)}\right\|}_{F}^{2}+\frac{1}{6}\sum_{i=1}^{3}\beta_{i}{\left\|Y_{i}-{\tilde{T}}_{\left(i\right)}\right\|}_{F}^{2}+\frac{1}{3}\sum_{i=1}^{3}\gamma_{i}{\left\|Y_{i}\right\|}_{*}$$

where $\gamma_{i}$ (different regularization parameter) and $\alpha_{i},\beta_{i}$ (different weights coefficient) can control the weight of each factor matrices so as to obtain accurate low rank components. Thus, the nuclear norm heuristics of the tensor CPD has been successfully established. Equation (14) is a typical convex optimization problem and we introduce an effective algorithm to solve this problem.

#### 2.2.3. The Working Algorithm

Many variables need to be solved: ${\tilde{T}}_{i},Y_{i},A_{i},\;i\;=\;1,2,3$, where the calculation of $Y_{i}$ is the main challenge. The alternating iterative decline method can be utilized to solve this optimization problem via calculating one variable with others fixing. Eventually, a global optimal solution of Equation (14) can be obtained.

**Computing** ${\tilde{T}}_{i}$**:** *When the other variables are fixed, the optimization problem will be simplified as*

(15) $$\underset{A_{i}}{\min}\frac{1}{6}\sum_{i=1}^{3}\beta_{i}{\left\|Y_{i}-{\tilde{T}}_{i}\right\|}_{F}^{2}.$$

The solution can be written as

(16) $${\tilde{T}}_{i}={\left(\frac{\sum_{j=1}^{3}\beta_{j}y_{j}}{\sum_{j=1}^{3}\beta_{j}}\right)}_{\left(i\right)},\;i=1,2,3.$$

**Computing** $A_{i}$**:** *The optimal* $A_{i}$ *can be obtained by Equation (12):*

(17) $$A_{1}={\tilde{T}}_{\left(1\right)}{\left[{\left(A_{2}⊙A_{3}\right)}^{T}\right]}^{†},A_{2}={\tilde{T}}_{\left(2\right)}{\left[{\left(A_{3}⊙A_{1}\right)}^{T}\right]}^{†},A_{3}={\tilde{T}}_{\left(3\right)}{\left[{\left(A_{2}⊙A_{1}\right)}^{T}\right]}^{†}.$$

**Computing** $Y_{i}$**:** *Similarly, the optimal* $Y_{i}$ *can be obtained by solving the following equation:*

(18) $$\frac{1}{3}\underset{Y_{i}}{\min}:\frac{1}{2}\alpha_{i}{\left\|Y_{i}-T_{\left(i\right)}\right\|}_{F}^{2}+\frac{1}{2}\beta_{i}{\left\|Y_{i}-{\tilde{T}}_{\left(i\right)}\right\|}_{F}^{2}+\gamma_{i}{\left\|Y_{i}\right\|}_{*}.$$

The singular value threshold [26] is employed to solve Equation (18), which is defined as follows:

(19) $$\Theta_{\lambda }(X)=U\sum {}_{\lambda }V^{T}$$

where $\sum {}_{\lambda }=diag(\max((\sigma_{i}-\lambda ),0))$. The literature [33] has proved that the optimal solution of Equation (20) can be shown as Equation (21).

(20) $$\underset{X}{\min}:\;\sum_{q=1}^{Q}\frac{\tau_{q}}{2}{\left\|X-D_{q}\right\|}_{F}^{2}+\gamma {\left\|X\right\|}_{*}$$

(21) $$X^{*}=\Theta_{\lambda }(D)$$

where $\lambda =\frac{\gamma }{\sum_{q=1}^{Q}\tau_{q}}$ and $D=\frac{1}{\sum_{q=1}^{Q}\tau_{q}}\sum_{q=1}^{Q}\tau_{q}D_{q}$.

Therefore, the optimal solution of Equation (18) can be solved by

(22) $$Y_{i}^{*}=\Theta_{\lambda_{i}}({\hat{Y}}_{i})$$

where $\lambda_{i}=\frac{\gamma_{i}}{\alpha_{i}+\beta_{i}}$, ${\hat{Y}}_{i}=\frac{\alpha_{i}T_{\left(i\right)}+\beta_{i}{\tilde{T}}_{\left(i\right)}}{\alpha_{i}+\beta_{i}}$. Thus, we can obtain the global optimal low-rank factor matrices, as well as the low-rank approximate tensor, which displays the characteristic information of the original signal data. The flowchart of the entire algorithm is shown in Algorithm 1.

**Algorithm 1: TNNCPD****Input:**
T **Output:**
$\tilde{T},\;A_{i},\;Y_{i},\;i=1,2,3$ **1: Initialize:**
$\tilde{T}=T,$
$A_{i}=$ R order vectors in front of the of $T_{\left(i\right)}$; The largest number of iterations: n=1000; convergence threshold: η=0.001; **2: while no convergence do** **3:  for**
i=1 to 3 do **4:   **
$Y_{i}=\Theta_{\frac{\gamma_{i}}{\alpha_{i}+\beta_{i}}}(\frac{\alpha_{i}T_{\left(i\right)}+\beta_{i}{\tilde{T}}_{\left(i\right)}}{\alpha_{i}+\beta_{i}})$ **5:  end for**
i=1 to 3 do **6:**   ${\tilde{T}}_{i}={\left(\frac{\sum_{j=1}^{3}\beta_{j}y_{j}}{\sum_{j=1}^{3}\beta_{j}}\right)}_{\left(i\right)}$ **7:  end for**  $A_{1}={\tilde{T}}_{\left(1\right)}{\left[{\left(A_{2}⊙A_{3}\right)}^{T}\right]}^{†},A_{2}={\tilde{T}}_{\left(2\right)}{\left[{\left(A_{3}⊙A_{1}\right)}^{T}\right]}^{†},A_{3}={\tilde{T}}_{\left(3\right)}{\left[{\left(A_{2}⊙A_{1}\right)}^{T}\right]}^{†}$ **8: end**

#### 2.2.4. Simulation Analysis

**Figure 3:**
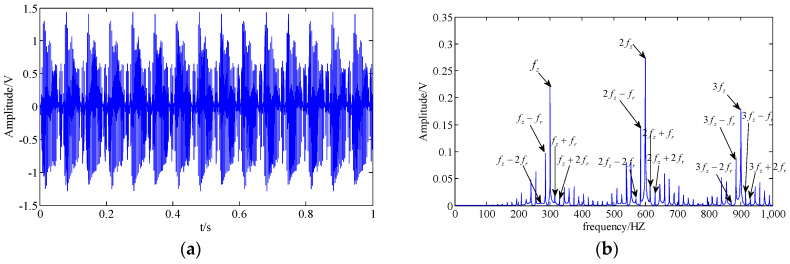

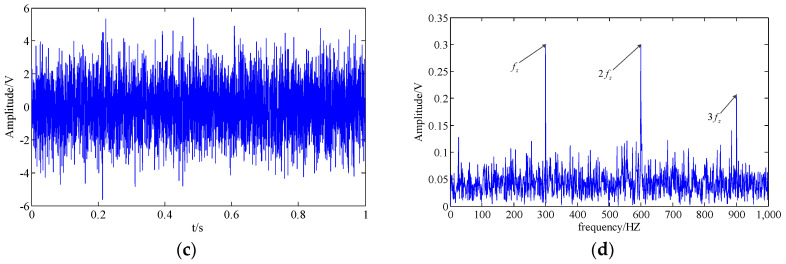
Simulation signal; (**a**,**b**) clean original signal in the time domain and the frequency domain; (**c**,**d**) original signal with noise in the time domain and the frequency domain.

Gears are essential components in rotation machinery and their vibration signals generally consist of multi-components, where the meshing frequencies of each pair of gears are the dominant characteristic [5]. Consider a pair of gears that mesh at a constant speed and constant load. When faults occur in the gear, for instance, the first gear, the meshing frequency, and its multiple frequencies are modulated by the rotational frequency of the first gear. Theoretically, the tooth meshing vibration function can be represented as [34]:

(23) $$y(t)=\sum_{m=0}^{M}Y_{m}(1+a_{m}(t))\cos(2\pi mf_{z}t+\phi_{m}+b_{m}(t))$$

(24) $$a_{m}(t)=\sum_{n=0}^{N}A_{mn}\cos(z\pi nf_{r}t+\alpha_{mn})$$

(25) $$b_{m}(t)=\sum_{n=0}^{N}B_{mn}\cos(z\pi nf_{r}t+\beta_{mn})$$

where M is the number of tooth meshing harmonics, $f_{z}$ is the tooth mesh frequency, $Y_{m}$ and $\phi_{m}$ represent the amplitude and the phase of the m-th meshing harmonics, $a_{m}$ and $b_{m}$ are the amplitude modulation function and phase modulation function, respectively, $f_{r}$ is the rotational frequency of the first gear, N is the number of pairs of modulation sidebands, and $A_{mn}$ and $B_{mn}$ as well as $\alpha_{mn}$ and $\beta_{mn}$ represent the amplitude and the phase. Thus, we create a gear simulation signal as follows:

(26) $$\begin{array}{cc} y(t)= & 0.2(1+a_{m}(t))\cos(2\pi \times 300t+10+b_{m}(t))\\ & +0.3(1+a_{m}(t))\cos(2\pi \times 600t+20+b_{m}(t))\\ & +0.2(1+a_{m}(t))\cos(2\pi \times 900t+40+b_{m}(t))+n(t) \end{array}$$

(27) $$a_{m}(t)=0.4\cos(2\pi \times 15t+10)+0.4\cos(2\pi \times 30t+20)+0.4\cos(2\pi \times 45t+40)$$

(28) $$b_{m}(t)=0.4\cos(2\pi \times 15t+20)+0.4\cos(2\pi \times 30t+40)+0.4\cos(2\pi \times 45t+60)$$

where n(t) is the Gaussian white noise with a variance of 1.5. The sampling frequency and sampling points are set as 4000 Hz and 4000. The clean original signal in the time domain and frequency domain are shown in Figure 3a,b, respectively. Additionally, the original signal with noise is shown in Figure 3c,d.

The modulation sidebands are masked by the strong background noise, and the fault feature is difficult to recognize. The TNNCPD method is employed to extract the useful feature component of the signal, while the SSA, WT, and CPD-ALS methods are selected for comparative analysis.

**Figure 4:**
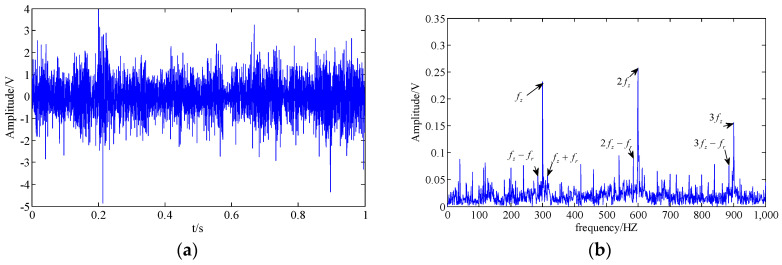
Results obtained by the singular spectrum analysis (SSA) method; (**a**,**b**) extracted feature component in the time domain and the frequency domain respectively.

**Figure 5:**
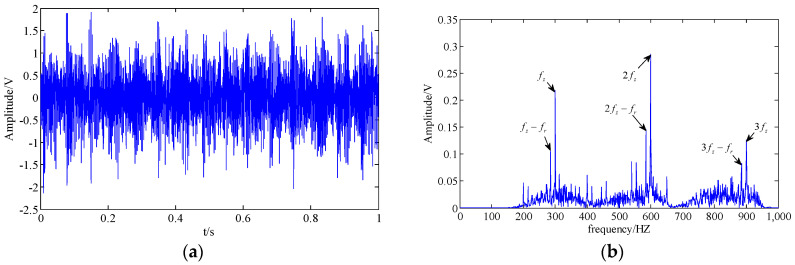
Result obtained by the wavelet transform (WT) method; (**a**,**b**) extracted feature component in the time domain and the frequency domain.

The results of the SSA and WT methods are shown in Figure 4 and Figure 5. In the SSA, we select characteristic components to construct the final time series by the hard threshold method [35] with a singular value threshold to retain 95%. In Figure 4b, the tooth meshing harmonics ($f_{z},2f_{z},3f_{z}$) and some of their modulation sidebands ($f_{z}\pm f_{r},2f_{z}-f_{r},3f_{z}-f_{r}$) can be found. However, the fault feature is still not easy to be identified because the modulation sidebands are incomplete. Following the method of WT, we select the layer number of wavelet decomposition as 5 and the basis function as db15. Analogously, the modulation sidebands in Figure 5 are incomplete as well.

**Figure 6:**
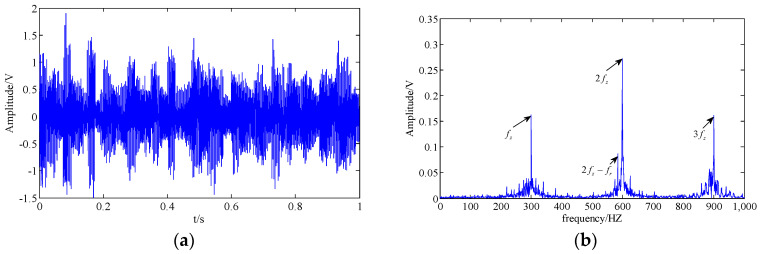
Result obtained by the least squares algorithm for CPD (CPD-ALS) method; (**a**,**b**) extracted feature component in the time domain and the frequency domain.

Subsequently, the CPD-ALS method is utilized to extract the feature information of the simulation signal. The rank of the tensor is chosen as 10, so ten rank-1 sub-tensors can be obtained by the CPD-ALS algorithm. Then, the correlation coefficient between each sub-tensors and the original signal is calculated, and some sub-tensors whose coefficient is larger (for instance, the top five sub-tensors) is reconstructed as the one-dimensional time series representing the extracted feature component of the signal. Results are shown in Figure 6. The modulation sidebands have almost disappeared in Figure 6b. Obviously, we cannot judge the fault condition of the simulation signal.

**Figure 7:**
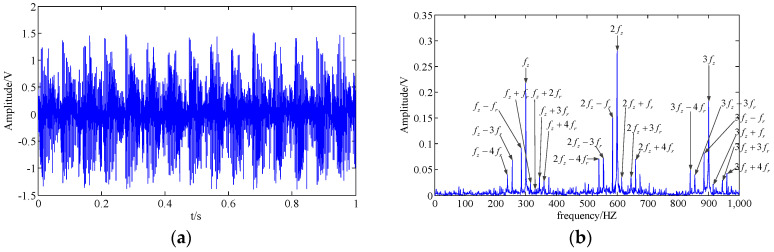
Result obtained by the proposed method; (**a**,**b**) extracted feature component in the time domain and the frequency domain.

The proposed method is employed to address the simulation signal. Firstly, the original simulation signal is analyzed using the TNNCPD method, so we can obtain a low-rank approximate tensor, which consists of three factor matrices representing a feature component of the original signal. Then, we reconstruct the low-rank approximate tensor as a one-dimensional time series, the result of which is shown in Figure 7. The tooth meshing harmonics ($f_{z},2f_{z},3f_{z}$) and almost all of their modulation sidebands are very clear. Consequently, the fault feature of the simulation signal has been successfully extracted via the TNNCPD method. The numerical simulation results show that the performance of the TNNCPD method is clearly superior to the three above-mentioned alternatives.

To allow for a more direct evaluation of the four methods’ performance, Table 1 reveals the correlation coefficient between the extracted results obtained using the above four methods and the original signal without noise. The results of the proposed method are closer to the original signal without noisy components, explicating that the performance of the proposed method outperforms the three alternative methods.

## 3. A Joint Fault Diagnosis Scheme Based on TNNCPD and MSPE

After the fault feature component of the measured vibration signal is extracted via TNNCPD, the fault patterns need to be identified. Multi-scale permutation entropy (MSPE) [28] can evaluate the complexity of signal in different scales by measuring the relative frequencies of different ordinal patterns. Therefore, MSPE can be chosen as the recognizer of different signal fault patterns. Thus, we proposed a joint fault diagnosis scheme based on TNNCPD and MSPE to perform gear fault classification and a detailed description of the method is as follows:

(1) Vibration signals reflecting the different working conditions of gears are acquired through the vibration acceleration sensor.
(2) The vibration signals are decomposed by the TNNCPD method to obtain the useful feature component.
(3) The MSPE of the feature component is calculated as a fault feature vector. The calculation process of MSPE is as follows:

Firstly, the fault feature time series $x=[x_{1},x_{2},\ldots ,x_{N}]$ is transformed into a successive coarse-grained time series $y^{\left(\varepsilon \right)}$ with a scale factor ε:

(29) $$y^{\varepsilon }{}_{j}=\frac{1}{\varepsilon }\sum_{k=(j-1)\varepsilon +1}^{j\varepsilon }x_{k},\;j=1,2,\ldots ,[N/\varepsilon ].$$

The coarse-grained time series are then converted into a series of data segments through the predetermined embedding dimension d and time-lag τ:

(30) $$v^{n}=[y^{\varepsilon }{}_{n},y^{\varepsilon }{}_{n+\tau },\ldots ,y^{\varepsilon }{}_{n+(d-1)\tau }],\;n=1,2,\ldots ,N-(d-1)\tau .$$

There are d! possible ordinal patterns ($\varphi_{i},i=1,\ldots ,d!$) by arranging $v^{n}$ in increasing order of magnitudes. Let $f(\varphi_{i}),i=1,\ldots ,d!$ denote the frequency of its occurrence in the data segments, so that the relative frequency is thus

(31) $$p(\varphi_{i})=f(\varphi_{i})/(N-(d-1)\tau ).$$

Finally, the MSPE is defined as

(32) $$P(\varepsilon )=-\sum_{i=1}^{d!}p(\varphi_{i})\log_{2}p(\varphi_{i}).$$

For convenience, we normalize P(ε) by its maximum value $\log_{2}d!$:

(33) $$0\leq P(\varepsilon {)/\log}_{2}d!\leq 1.$$

**Figure 8:**
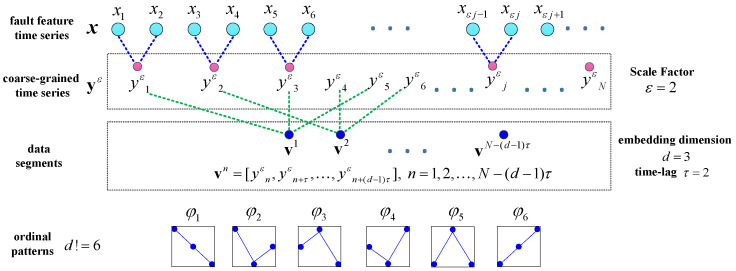
The scheme illustrating the coarse-graining and data segments of the fault feature time series with ε=2,d=3,τ=2.

Thus, we can obtain the fault feature vector P=[P(1),P(2),⋯]. Figure 8 illustrates the coarse-graining and data segments of the fault feature time series.

**Figure 9:**
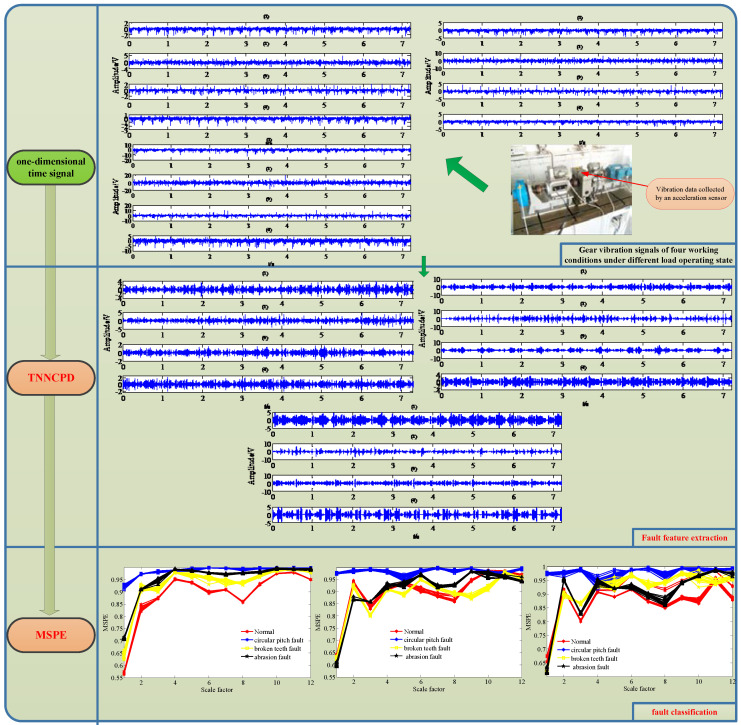
The flowchart of the proposed joint fault diagnosis scheme based on tensor nuclear norm canonical polyadic decomposition (TNNCPD) and multi-scale permutation entropy (MSPE).

(4) Enter the fault feature vector as the input of the BP neural network chosen fault classifier in order to achieve fault classification. The flowchart of the proposed method is depicted in Figure 9.

## 4. Experimental Signal Analysis

**Figure 10:**
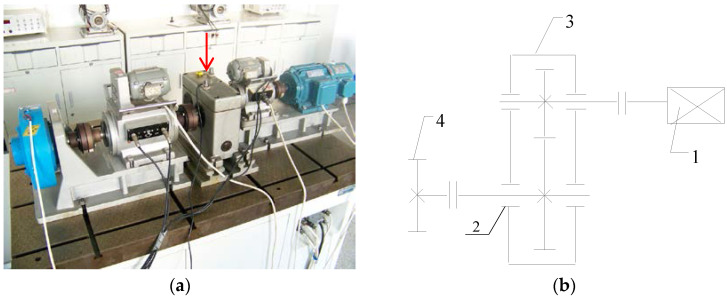
Experimental device. (**a**) Device image. (**b**) Device structure diagram: 1—AC motor; 2—measurement point; 3—gearbox; 4—magnetic powder brake.

In order to verify the effectiveness of the proposed joint fault diagnosis, we collected the gear vibration signals of different working conditions for analysis, including normal condition, circular pitch fault, broken teeth fault, and abrasion fault. The experimental device is demonstrated in Figure 10. It consists of an AC motor, couplings, dynamometers, a gearbox with a pair of meshing gears, and a magnetic powder brake. The red arrow indicates the acceleration vibration sensor placement. The number of teeth on the pinion and wheel are 20 and 37, and the gear modulus is equal to 3. The spindle speed is maintained at 1154 r/min, while the data sampling frequency is 2000 Hz. We have tested three operating states of different loads for each gear working condition, including a no-load operating state, an operating state with a load of 10 N/m, and an operating state with a load of 20 N/m. Each working condition under operating states with different loads contains 19 group data samples, so there are 76 group data samples in total for each operating state, and each data sample has 15,000 data points.

**Figure 11:**
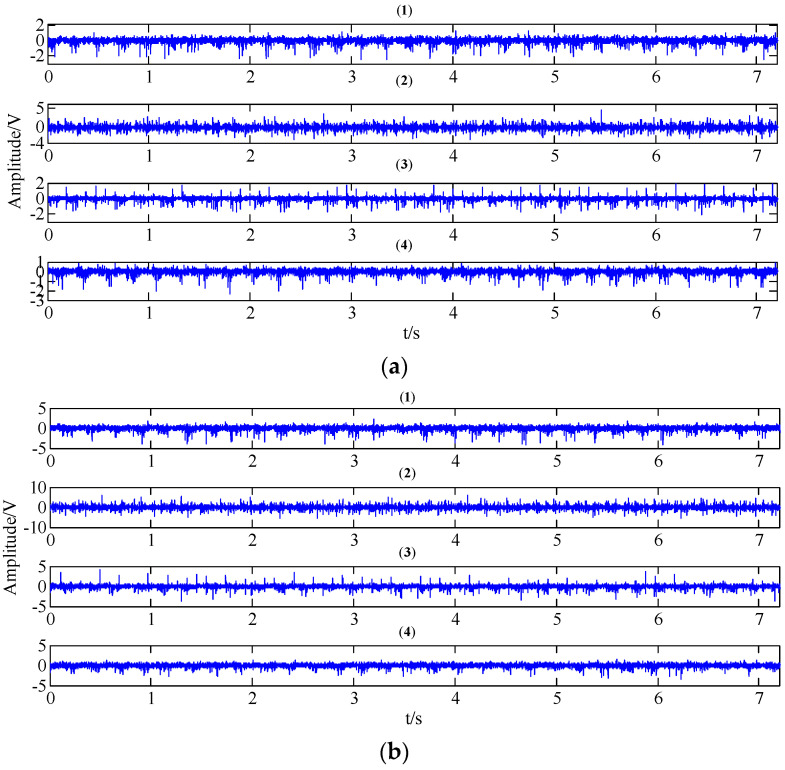

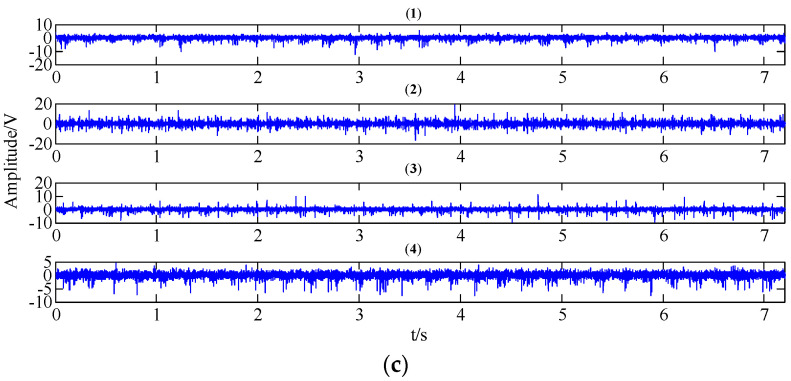
The collected gear vibration signals of four working conditions ((**1**) normal condition; (**2**) circular pitch fault; (**3**) broken teeth fault; (**4**) abrasion fault) under different operating state loads in the time domain; (**a**) no-load operating state; (**b**) an operating state with a load of 10 N/m; (**c**) an operating state with a load of 20 N/m.

Figure 11 reveals the collected vibration signals of four gear working conditions under different operating state loads in the time domain. It can be seen that these signals show strong non-linear and non-stationary features. Moreover, the features and amplitudes of the normal condition, the circular pitch fault condition, and the abrasion fault condition are very similar, so it is not easy to distinguish these four working conditions.

**Figure 12:**
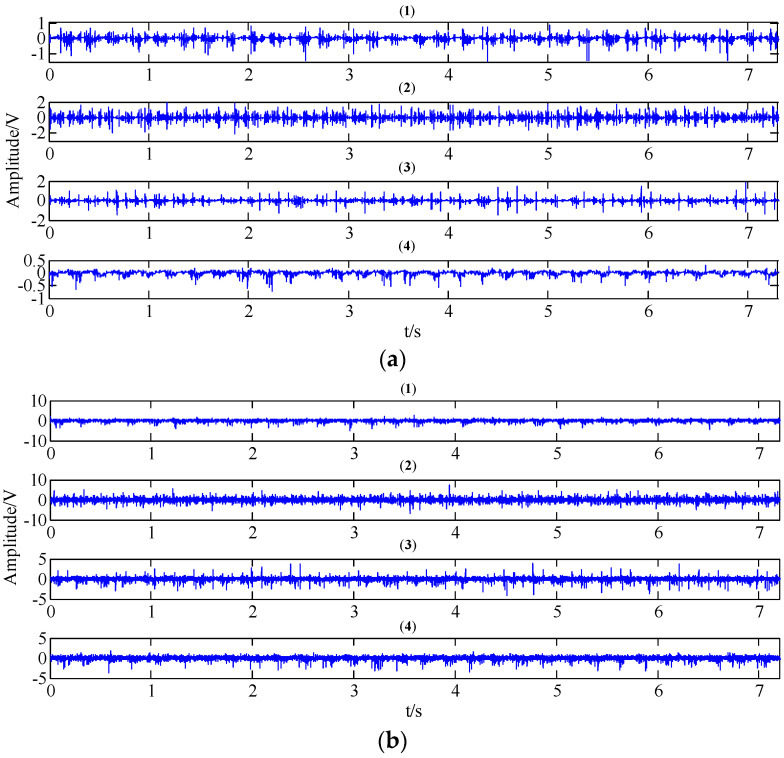

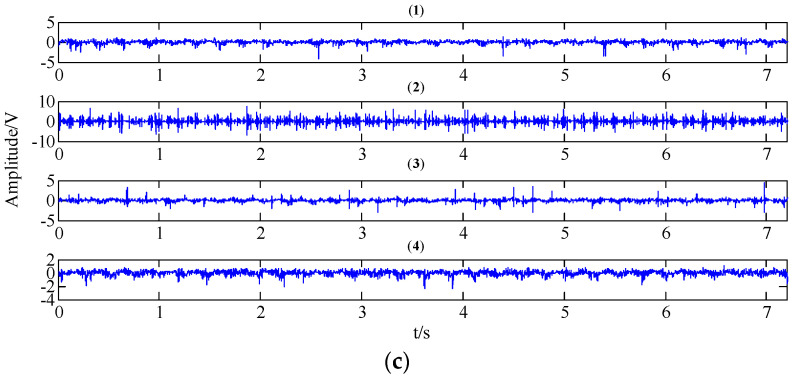
The feature extracted results of the gear signals of four working conditions ((**1**) normal condition; (**2**) circular pitch fault; (**3**) broken teeth fault; (**4**) abrasion fault) under different operating state loads in the time domain by the WT method; (**a**) no-load operating state; (**b**) an operating state with a load of 10 N/m; (**c**) an operating state with a load of 20 N/m.

**Figure 13:**
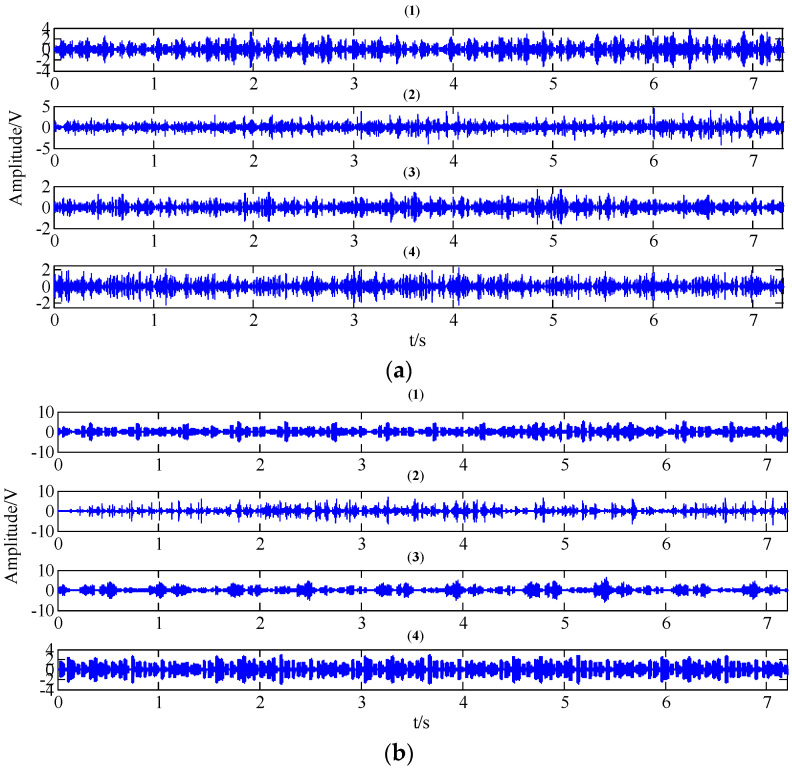

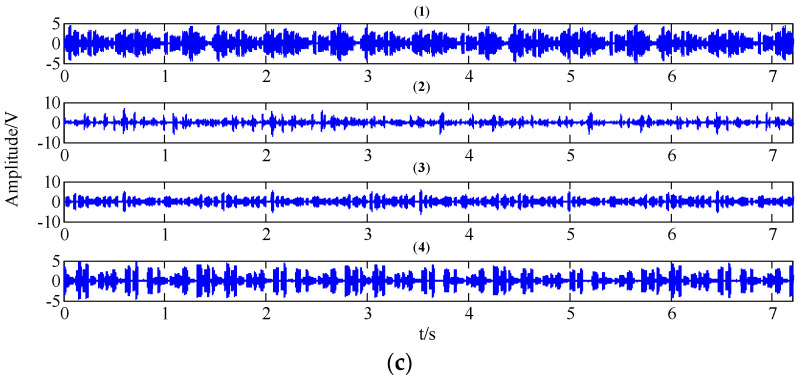
The feature extracted results of the gear signals of four working conditions ((**1**) normal condition; (**2**) circular pitch fault; (**3**) broken teeth fault; (**4**) abrasion fault) under different operating state loads in the time domain by the TNNCPD method; (**a**) no-load operating state; (**b**) an operating state with a load of 10 N/m; (**c**) an operating state with a load of 20 N/m.

The WT and the TNNCPD methods were used to extract the feature component of the four working condition signals. The computed results are plotted in Figure 12 and Figure 13, respectively. From the two figures, we can inspect that the extracted feature components of different working states show different characteristics, mainly including different periodicities and amplitudes.

**Figure 14:**
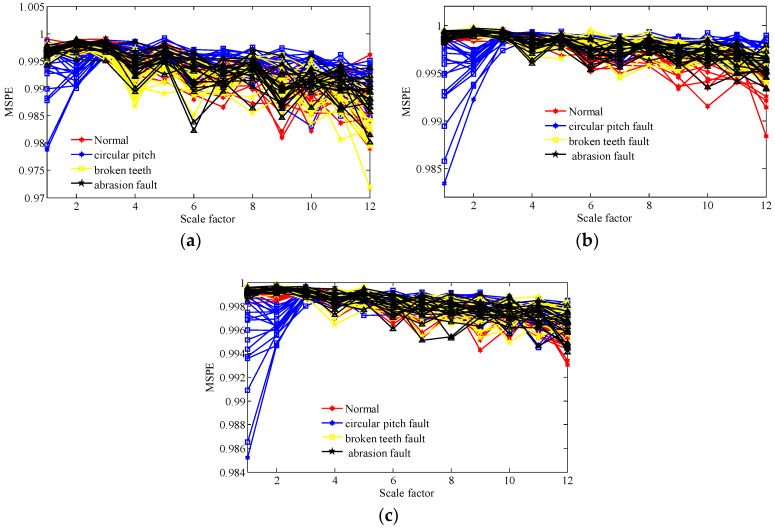
MPSE of over 12 scales of all the samples of original gear signals of four working conditions under different operating state loads; (**a**) no-load operating state; (**b**) an operating state with a load of 10 N/m; (**c**) an operating state with a load of 20 N/m.

**Figure 15:**
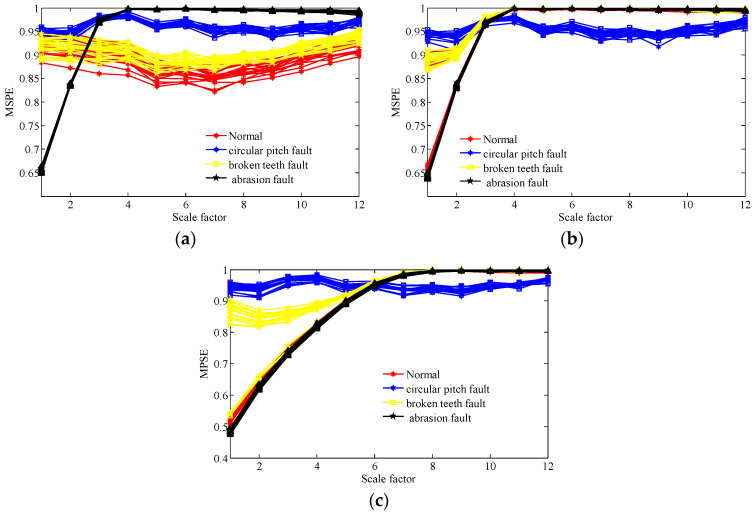
MPSE of over 12 scales of all samples of extracted feature components of four working conditions under different operating state loads by the WT method; (**a**) no-load operating state; (**b**) an operating state with a load of 10 N/m; (**c**) an operating state with a load of 20 N/m.

**Figure 16:**
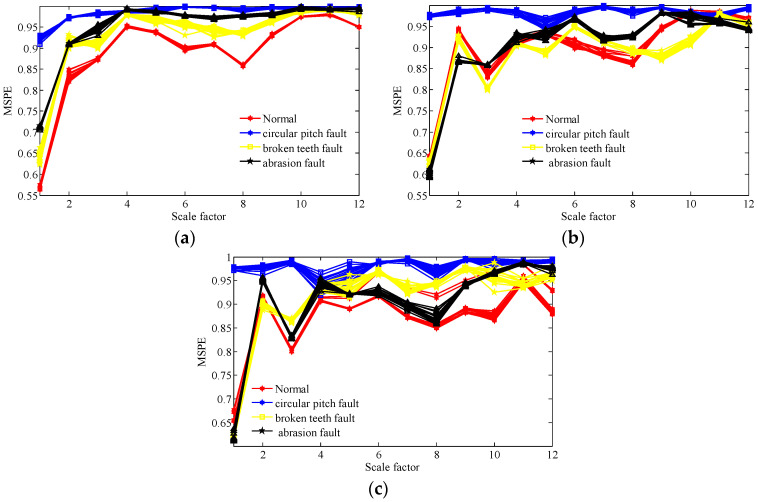
MPSE of over 12 scales of all samples of extracted feature components of four working conditions under different operating state loads by the TNNCPD method; (**a**) no-load operating state; (**b**) an operating state with a load of 10 N/m; (**c**) an operating state with a load of 20 N/m.

Then, we calculate the MPSE of the obtained feature components via the two methods and the original signals. The parameters of the MPSE are set as m=4,τ=2 and the value range of the scale factor is determined as ε=1−12. The computed results are displayed in Figure 14, Figure 15 and Figure 16.

Figure 14 shows that the MSPE of all samples of original gear signals under different operating state loads are almost mixing together. Therefore, it is difficult to immediately recognize the working condition from the original signals. From Figure 15, we can see that the MSPE of the normal condition, broken teeth fault, and abrasion fault remain mixed together, therefore, making it challenging to differentiate between the three kinds of faults. As such, the WT method may possess some deficiencies in terms of dealing with the measured faulty gear signals.

From Figure 16, it is evident that the lines corresponding to the four working conditions under different operating state loads can be separated in many scales. The analysis results demonstrate that the proposed scheme not only has a good fault feature extraction effect for the gear signals, but also can effective identify the working condition of different fault feature components.

**Figure 17:**
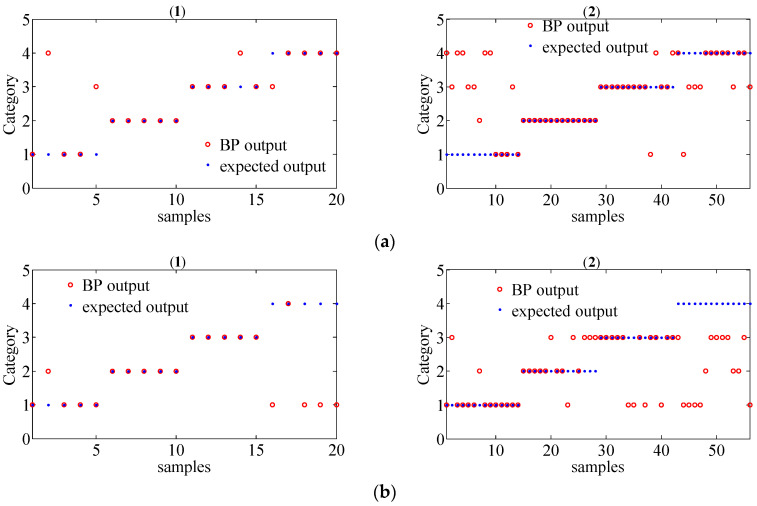

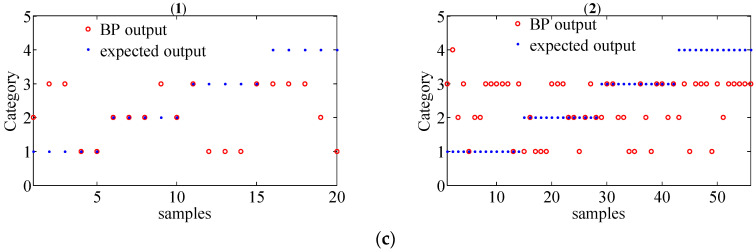
The classification results of gear signals of four working conditions under different operating state loads by the MSPE ((1) train set; (2) test set); (**a**) no-load operating state; (**b**) an operating state with a load of 10 N/m; (**c**) an operating state with a load of 20 N/m.

**Figure 18:**
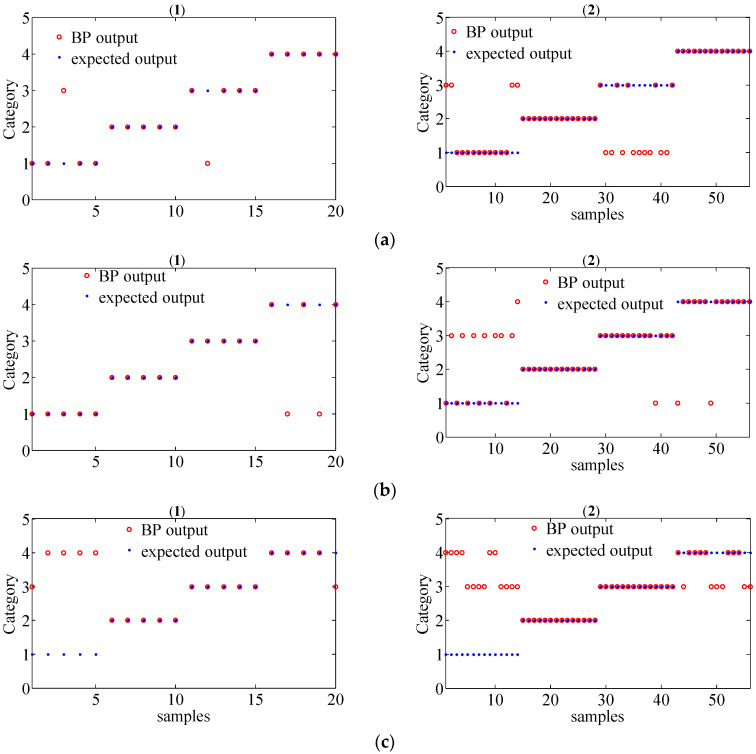
The classification results of gear signals of four working conditions under different operating state loads by combining WT with MSPE method ((1) train set; (2) test set); (**a**) no-load operating state; (**b**) an operating state with a load of 10 N/m; (**c**) an operating state with a load of 20 N/m.

**Figure 19:**
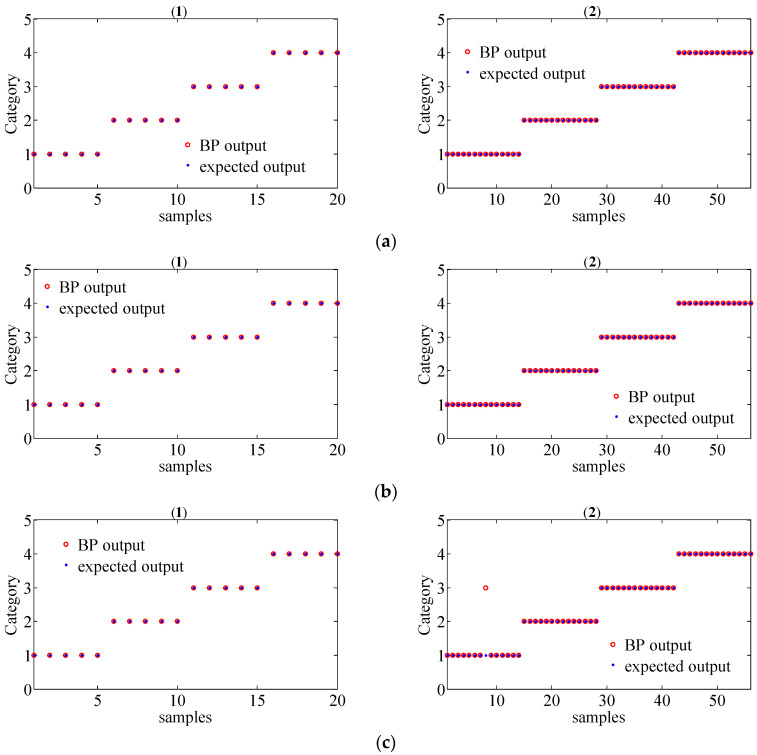
The classification results of gear signals with four working conditions under different operating state loads by the proposed joint fault diagnosis scheme ((1) train set; (2) test set); (**a**) no-load operating state; (**b**) an operating state with a load of 10 N/m; (**c**) an operating state with a load of 20 N/m.

In order to further evaluate the performance of the proposed method for gear fault diagnosis, the MSPE vectors were chosen as the inputs of the BP neural network to achieve fault classification. The parameter of the classifier was set as follows: the input layer has 12 nodes, a hidden layer has 25 nodes, and the output layer has 4 nodes. Since there are 19 group data samples in each working condition for different operating state loads, we randomly selected 5 group samples as the training set and the remaining 14 group samples as the test set. Thus, there are 20 group train set and 56 group test train set in total for different operating state loads. The classification results of the original signals by the MSPE method, by combining WT with the MSPE method and by the proposed joint fault diagnosis scheme, are shown in Figure 17, Figure 18 and Figure 19, respectively. The category labels 1, 2, 3, and 4 represent normal conditions, circular pitch faults, broken teeth faults, and abrasion faults, respectively. Obviously, for the original signals, all four working conditions are difficult to distinguish. In Figure 18, the separation effect of the normal condition, broken teeth fault, and circular pitch fault is suboptimal. As shown in Figure 19, each gear working state was recognized. Table 2 lists the classification accuracy of each method, it is very clear that the accuracy of the proposed scheme is close to 100%, indicating that its performance is significantly better than the MSPE method and combining WT with the MSPE method. The classification results show that the proposed joint fault diagnosis scheme has excellent classification performance for different gear faults.

## 5. Conclusions

A joint fault diagnosis scheme based on tensor nuclear norm canonical polyadic decomposition (TNNCPD) and multi-scale permutation entropy (MSPE) has been proposed for gear fault classification. The main contributions of this paper are as follows: (1) A new tensor canonical polyadic decomposition method based on nuclear norm and convex optimization is established, and an efficient working algorithm is provided. (2) The proposed TNNCPD method and MSPE method is combined to achieve gear fault classification, in which the characteristic components of the original signals can be extracted via the TNNCPD method, and the MPSE of the characteristic components has been calculated as the feature vector to recognize fault conditions. (3) The proposed scheme has been applied in different gear fault classifications under different operating state loads. The proposed scheme demonstrated excellent fault classification performance. It needs to be pointed out, however, that the calculation time of the scheme is a problem. Since the TNNCPD converts the one-dimensional time series into three-dimensional data, which inevitably leads to an increased amount of computing data, the calculation time of the TNNCPD is longer than that of the other methods based on vectors or matrix models. Thus, how the calculation efficiency of the TNNCPD method can be effectively improved will be one of the highlights of our next research work.

## Figures and Tables

**Table 1:** The correlation coefficient between the feature component obtained by the four methods and the source signal without noisy components.

	SSA	WT	CPD-ALS	The Proposed Method
correlation coefficient	0.8142	0.8352	0.7051	0.9351

SSA: singular spectrum analysis; WT: wavelet transform; CPD-ALS: least squares algorithm for canonical polyadic decomposition.

**Table 2:** The classification accuracy of each method.

Operating State	Set	MSPE	Combining WT with MSPE	Proposed Scheme
no load	train	80%	90%	100%
	test	66.07%	76.79%	100%
a load of 10 N/m	train	75%	90%	100%
	test	53.57%	80.36%	100%
a load of 20 N/m	train	40%	70%	100%
	test	23.21%	64.28%	98.21%

MSPE: multi-scale permutation entropy.
